# How has the COVID-19 epidemic affected the risk behaviors of people who inject drugs in a city with high harm reduction service coverage in Vietnam? A qualitative investigation

**DOI:** 10.1186/s12954-021-00586-1

**Published:** 2022-01-29

**Authors:** Trang Thu Nguyen, Giang Thi Hoang, Duc Quang Nguyen, Anh Huu Nguyen, Ngoc Anh Luong, Didier Laureillard, Nicolas Nagot, Don Des Jarlais, Huong Thi Duong, Thanh Thi Tuyet Nham, Oanh Thi Hai Khuat, Khue Minh Pham, Mai Sao Le, Laurent Michel, Delphine Rapoud, Giang Minh Le

**Affiliations:** 1 Centre for Training and Research on Substance Use and HIV, Hanoi Medical University, Room 211B, Building E3, #1 Ton That Tung Street, Hanoi, Vietnam; 2 Hai Phong University of Medicine and Pharmacy, Haiphong, Vietnam; 3 Pathogenesis and Control of Chronic and Emerging Infections, INSERM, Etablissement Français du Sang, University of Montpellier, Montpellier, France; 4 Infectious Diseases Department, Caremeau University Hospital, Nîmes, France; 5 New-York University College of Public Health, New York, NY USA; 6 Supporting Community Development Initiatives, Hanoi, Vietnam; 7 CESP Inserm UMRS 1018, Paris Saclay University, Pierre Nicole Center, French Red Cross, 27 rue Pierre Nicole, 75005 Paris, France

**Keywords:** COVID-19, Risk behaviors, People who inject drugs, Harm reduction

## Abstract

**Introduction:**

The COVID-19 outbreak disproportionally affects vulnerable populations including people who inject drugs (PWID). Social distancing and stay-at-home orders might result in a lack of access to medical and social services, poorer mental health, and financial precariousness, and thus, increases in HIV and HCV risk behaviors. This article explores how the HIV/HCV risk behaviors of PWID in Haiphong, a city with high harm reduction service coverage in Vietnam, changed during the early phase of the COVID-19 pandemic, and what shaped such changes, using the risk environment framework.

**Method:**

We conducted three focus group discussions with peer outreach workers in May 2020 at the very end of the first lockdown, and 30 in-depth interviews with PWID between September and October 2020, after the second wave of infection in Vietnam. Discussions and interviews centered on the impact of the COVID-19 pandemic on their lives, and how their drug use and sexual behaviors changed as a result of the pandemic.

**Results:**

The national shutdown of nonessential businesses due to the COVID-19 epidemic caused substantial economic challenges to participants, who mostly were in a precarious financial situation before the start of the epidemic. Unsafe injection is no longer an issue among our sample of PWID in Haiphong thanks to a combination of different factors, including high awareness of injection-related HIV/HCV risk and the availability of methadone treatment. However, group methamphetamine use as a means to cope with the boredom and stress related to COVID-19 was common during the lockdown. Sharing of smoking equipment was a standard practice. Female sex workers, especially those who were active heroin users, suffered most from COVID-related financial pressure and may have engaged in unsafe sex.

**Conclusion:**

While unsafe drug injection might no longer be an issue, group methamphetamine use and unsafe sex were the two most worrisome HIV/HCV risk behaviors of PWID in Haiphong during the social distancing and lockdown periods. These elevated risks could continue beyond the enforced lockdown periods, given PWID in general, and PWID who are also sex workers in particular, have been disproportionately affected during the global crisis.

## Introduction

The current outbreak of the novel coronavirus SARS-CoV-2 (COVID-19) has spread all over the world since the first case was detected in December 2019. The pandemic poses a serious threat to global health with significant loss of life and the impact on the society as a whole through the mitigation measures taken to control it. It disproportionately affects vulnerable populations, including people who inject drugs (PWID), of which the majority live in poor socioeconomic conditions [1].

Existing literature points to multiple factors at different levels that may contribute to the harms PWID currently experience during the COVID-19 pandemic [2–6]. People who use drugs are particularly vulnerable to SARS-CoV-2 infection and complications due the high prevalence co-morbidities in their population [5–8]. Many have lung and liver diseases that may dangerously contribute to the aggravation of COVID-related disease [3]. Moreover, the high prevalence of HIV infection in this population indicates that some have weakened immunity and low resistance to COVID infection [3, 7]. A large number of people who use drugs also lack access to personal protection measures such as private space to effectively self-isolate [9]. Job loss or reduced income might cause them great challenges, especially those living hand-to-mouth [1]. Restrictions on face-to-face contact, reduced number of visits per day, or even temporary closures of harm reduction services might result in a lack of timely support for PWID, thus increasing HIV and HCV risk behaviors [3, 10, 11]. Lastly, social isolation and stress might exacerbate negative effects on their mental health [3, 12].

The link between the COVID-19 pandemic and HIV/HCV risk behaviors is little studied. While traditional risk factors like injection equipment sharing and unsafe sexual behaviors might decrease as a result of physical distancing and curfew implementation in many countries [13], these measures might also introduce new risks. A study showed that while sexual risk behaviors among sexual minority men overall reduced during the COVID-19 pandemic, such behaviors increased among those who use drugs [14]. A report from Thailand also suggested increase in sexualized drug use among gay and bisexual men which could lead to sexual risk behaviors [15]. People might also increase their consumption of alcohol and other drugs to cope with the stress related to the pandemic, or get involved with informal economies like sex work to overcome financial challenges [3, 5, 10]. As COVID-19 public health responses are stretching the already limited resources for the prevention and treatment of drug-related issues, a good understanding of the situation is critical to implement cost-effective harm reduction strategies.

Injection drug use, and more recently unsafe sex, remain the drivers of the HIV/HCV epidemic in Vietnam [16]. Harm reduction programs have been implemented in the country since the mid-1990s, as HIV prevalence among PWID rocketed from 10.1% in 1996 to 32% in 2002 [17]. Major strategies included the provision of sterile needles and syringes, condom distribution and methadone maintenance treatment [18]. Over more than a decade, methadone treatment has expanded from two clinics serving nearly 1000 patients in 2008, to 316 clinics in all provinces, serving about 53,000 (28%) people who use opioids in the country [19]. These programs reduced HIV prevalence among PWID to 12.7% in 2019 [19]. However, while HIV incidence in this group has decreased, their HCV incidence remains as high as 19.4/100 PY [20]. Moreover, increasing consumption of methamphetamine (hereafter: meth) among PWID, including methadone patients in recent years, is raising concerns about a resurgence of HIV/HCV infection and suboptimal treatment outcomes [19, 21–23].

The COVID-19 epidemic started in late January 2020 in Vietnam [24]. Despite the relatively small number of cases (254 by April 10th, 2020), Vietnam conducted the first nationwide lockdown for 3 weeks from April 1st, 2020 as an early response to the epidemic [25]. Nonessential businesses were shut down and medical professionals of different expertise were deployed to scale up COVID-19 prevention and treatment. Since then, subsequent lockdowns for extended periods have continued. Our study explores how the HIV and HCV risk behaviors of PWID changed during the COVID-19 pandemic in Haiphong—a socioeconomically important province in Northern Vietnam with a past drug injection-driven HIV epidemic—and what shaped such changes. At the time of the study, there were no confirmed COVID-19 cases in Haiphong. Nevertheless, the province was under the effect of national measures to contain the COVID-19 pandemic.

Since the early days of the HIV epidemic, the province has implemented various harm reduction strategies [26]. Haiphong is the study site of the 5-year DRIVE (DRug use and Infections in ViEtnam) project and its associated studies, which have aimed to prevent HIV/HCV transmission among PWID (heroin) in the province since 2016 [27]. This has included using a mass screening strategy coupled with HIV/HCV sensitization, harm reduction, and referral to HIV/HCV, methadone and psychiatric treatments by community-based outreach workers. In total, these studies have reached 4000 out of about 5000 active PWID in the province. Within these projects, 1000 PWID have been initiated on HCV treatment, and about 250 PWID presenting with a mental health diagnosis have received psychiatric treatment. Intensive harm reduction efforts have brought HIV incidence among PWID in Haiphong to a very low level, supporting the declaration of the end of the HIV epidemic among this population in the province [28]. The high number of PWID in HCV treatment also differentiates Haiphong from other provinces in terms of access to HCV care [29]. Given such achievements, Haiphong served as a good study context to understand the early impact of the COVID-19 pandemic on risk behaviors in PWID.

To investigate what influenced risk behaviors, we employed the risk environment framework [30]. This framework has gained popularity in public health over the last 2 decades. Risk environment can be defined as a ‘space—whether social or physical—in which a variety of factors interact to increase the chances of drug-related harms’ [30]. This framework emphasizes the role of contextual factors in producing or reducing drug-related harms, and provides an alternative to the biomedical and political perspectives that stress individual responsibility in managing harms [31, 32]. The risk environment framework delineates four types of environments—physical, social, political and economic—that interact among themselves and with the individual at micro and macro levels [30]. Identifying both risk and protective environmental factors, and the ways they shape PWID’s risk behaviors, is a straightforward way to foster ‘enabling environments’ for harm reduction [32].

## Method

This qualitative analysis is part of DRIVE-COVID, a mixed-methods research project that has investigated the changes in risk behaviors of PWID in Vietnam during the COVID-19 pandemic. We conducted three focus group discussions (FGDs) with peer outreach workers in May 2020 at the end of the first lockdown, and 30 in-depth interviews with PWID between September and October 2020, after the second wave of infection in the country. Peer outreach workers came from the three largest community-based organizations in Haiphong serving people who use drugs and female sex workers. We selected participants to cover a variety of characteristics including gender, age group, methadone treatment and HIV status, mental health treatment, and HCV treatment status. These characteristics have been reported in systematic reviews to have an association with the HIV/HCV risk behaviors [33–35].

The discussions with peer outreach workers centered on the impact of the COVID-19 pandemic on their lives, and their observation of changes in their clients’ injection and sexual risk behaviors. Interviews with participants who use drugs explored their experiences with the pandemic and the lockdown period, with a focus on how their drug use and sexual behaviors changed. The first author developed the FGDs and interview schedules from the research questions with inputs from the whole team. FGD and interviews were conducted in the offices of two community-based organizations that implement peer outreach. All FGDs and interviews were audio-recorded and transcribed verbatim. After being de-identified, transcripts were uploaded onto Atlas.ti version 8 [36] to facilitate coding. Participants received VND200,000 (~ US 9 dollars) for their time and effort. The study was approved by the Ethics Committee of Haiphong Medicine and Pharmacy University.

Our analysis was informed by the grounded theory approach. The authors at Hanoi Medical University conducted FGDs and interviews, and summarized salient themes as each session was completed. At first, we read the transcripts multiple times to immerse ourselves in participants’ stories. We then conducted open coding until no new codes emerged (after 5 transcripts) [37]. Examples of codes included ‘poor people die first’, ‘COVID-related financial constraints’, ‘challenging to purchase drugs’ or ‘care for clients during the lockdown’. We grouped the codes into larger categories of risk and protective environmental factors influencing risk behaviors. The qualitative findings were discussed within the study team and triangulated with the quantitative data on the description of participants’ risk behaviors.

## Results

Table 1 presents the participants’ sociodemographic characteristics. Women accounted for 57% of the sample. All participants self-identified as heterosexual. The median age of participants was 40.5 years. One third of participants were HIV-positive. Two thirds were unemployed or had unstable incomes. 60% were undergoing methadone treatment. Almost half of participants were actively using heroin (daily or once every several days) and one third were actively using meth (once every several days) at the time of the study.

**Table 1:** Participant characteristics (*N* = 30)

*Male/Female*	13/17
Transgendered people	0
Sex workers	5
*Age (median)*	40.5
*Education*	
Primary school	3
Middle school	10
High school & college	10
No information	7
*Occupation*	
Unemployed	7
Unstable income	13
Stable income	10
*Marital status*	
Single	7
Married/Cohabiting	12
Divorced/Widowed	11
*Received mental health treatment*	15
*Received HCV treatment*	10
*HIV positive/Under antiretroviral treatment*	9/9
*Currently in methadone treatment*	18
*Currently using heroin*	14
*Currently using meth*	10

The analysis centered on heroin injection, meth use in groups, and unsafe sexual behaviors as the key HIV/HCV risk behaviors among our participants. Participants reported no meth injection. The COVID-19 epidemic and containment policies introduced significant changes into the lives of participants. These changes exerted both negative and positive impacts on risk behaviors. Table 2 summarizes the risk and protective environmental factors influencing the three identified behaviors.

**Table 2:** Summary of risk and protective environmental factors for drug use and sexual behaviors during COVID-19 lockdown

Risk factors	Protective factors
***Heroin injection***	
**Social**	**Social**
Lockdown	High awareness of injection-related HIV/HCV risks
Fear of being arrested by the police when going to buy syringes or drugs	Ease of buying syringes and needles at pharmacies, even during the lockdown
**Economic**	Family support
Higher price of heroin	Social norms around heroin
	Hiding one’s drug use status
	**Policy**
	Availability of methadone treatment
	**Physical**
	Police presence in the street, curfew
	**Economic**
	Higher price of heroin
***Group methamphetamine use***	
**Social**	**Social**
Social norms related to meth use	Hiding one’s drug use status
**Physical**	**Economic**
Having more free time	Reduced income
Effects of meth	
**Sexual behaviors**	
**Social**	**Social**
Fear of COVID-19 infection	High awareness of HIV risk sexual behaviors
**Physical**	
Curfew, social distancing	
**Economic**	
Reduced income	

### Impact of the COVID-19 lockdown on participants’ lives and outreach work

While the lockdown could affect participants in all aspects of life, participants were most worried about its financial impact. All except one participant reported being challenged financially by the COVID-19 outbreak. Because of the lockdown, most socioeconomic activities ceased. Many participants with jobs like waste pickers, waiters, tea stall owners or security guards had to stay at home without work. Some lived hand-to-mouth and struggled to support themselves and their families. Factory workers, despite not losing their jobs, had to significantly reduce their working hours, and with it, their salaries. Few participants who had their ID card and were qualified as being ‘poor’ received some government support. But such support (about five kilograms of rice per person for 3 weeks of lockdown) was insufficient. A participant expressed her desperation:

It’s so stressful. You have no money. You can make no money. You can’t go out to steal; you can’t go out to sell sex. Everyone is dead. (Female, 42 years old, unemployed)

Another person highlighted that poor people were most vulnerable to the impacts of lockdown, noting that social inequality seems to be most pronounced in times of crisis:

For waste pickers, there is no junk to pick in COVID time […] For people who have money, during these 15, 20 days of lockdown, they could have some rest at home; for those without a decent job, they can make no money for food. (Female, 54 years old, waste picker)

Participants who were active users and were not already under methadone treatment were hit hardest. These people previously had jobs and relatively stable incomes to support their habit. However, as economic activities slowed down, their income also declined. All had to reduce their intake and in some way suffered the discomfort related to insufficient dosage. Participants expressed their anxiety when facing the inability to procure drugs:

When I saw on the news quarantined people [in another country] crying and screaming, I was so upset. In quarantine, money serves nothing. You won’t be able to buy drugs. [Sigh] You’re not addicted, you don’t know … (Female, 38 years old, sex worker)

Some participants reported that financial challenges caused family conflicts, and both were great stressors to them. Compared to the urgent concern for earning a living, the risk of COVID infection was not a priority. Indeed, many participants reported that they did not worry about contracting COVID while they had to endure hardship in their everyday lives.

#### Heroin injection

Among the 14 participants who were active heroin users at the time of the interview, 8 were not undergoing methadone treatment. Still, all of these active heroin users reported no sharing of injection equipment. This finding is consistent with our survey data, with 99.5% participants reported not having shared or divided drugs with a used needle or syringe over the last 6 months [38]. The protective social and policy factors against sharing of injection equipment included awareness of the HIV/HCV risks relating to unsafe injection, ease of buying equipment at pharmacies, family support and high methadone treatment coverage. A participant expressed her concerns about the HCV infection risk between her son and her. She reported how she instructed her son to use drugs safely:

I tell him to use a syringe once and that only when he runs out of money can he use it twice. Each time he would need to wash it with clean water, put it in a safe place and never share it with anyone. My HCV infection has been cured, his hasn’t. (Female, 54 years old, waste picker)

Even during the lockdown, participants were still able to buy sterile syringes at pharmacies. A slight increase in price in some pharmacies did not interfere with their practice. Participants often bought syringes for each day’s worth of use. Some family even assumed this task and bought boxes of syringes for participants so that they did not have to go out during the lockdown.

The safe injection practice could also have been fostered by the fact that some participants insisted on using heroin alone, since they did not want to disclose their heroin use to their drug using peers:

I’ve been doing heroin alone since I relapsed. I don’t want my friends to know about it. They know I’m doing meth but for them, heroin users are worse than meth users. (Male, 37 years old, unemployed)

Doing heroin alone, however, could increase the risk of overdose death [39].

During lockdown, some participants had to reduce their heroin intake because of their income reduction and the challenges in purchasing heroin. Some did not want their family members to know they were still using heroin. Participants undergoing methadone treatment encountered no difficulty in temporarily stopping or reducing their heroin consumption, while those not undergoing treatment struggled. One participant described the difficulties in buying drugs:

During the lockdown, the police were everywhere. Neither we nor the dealers were allowed to go out. It was too easy to get caught if you went to buy drugs. (Male, 27 years old, painter)

These challenges made him decide to get methadone treatment:

I’ve known about methadone programs for two years but I didn’t really pay attention to it. But given the last lockdown period, I made up my mind and got treatment. (Male, 27 years old, painter)

Outreach workers were, however, less confident that everyone could practice safe injection during the social distancing period and lockdown. They explained:

The dealers no longer sold drugs in small quantities. Several people had to pool their money to buy drugs together. However, since sharing dry drugs results in unequal partitioning, they’d mix drugs with water in a new syringe. Some without money would have to use others' needles. (FGD with peer outreach workers)

Thus, from the outreach workers’ perspective, the same financial challenges in buying drugs could facilitate unsafe injection.

Other participants also mentioned the prospect of being arrested by the police while going out to buy sterile syringes at pharmacies.

I know there are many cops around the pharmacy at Hospital X. They’d stay behind you. When they’d see you were buying syringes, they’d get you. That’s why I’ve never bought [syringes] there. (Female, 23 years old, assistant mason)

While there was no report of its impact on injection behaviors, this fear might play a role in the fact that some participants reused their own syringes to avoid going out too often, and thus increased their risk of bacterial infection [40].

### Group meth use

Most participants had experience using meth. Among them, 10 people were active meth users and 2 were HCV-positive. Participants often used meth in social gatherings and shared the same smoking tools (bongs and pipes). Only two participants reported doing meth exclusively by themselves. The tools were not disinfected after each use and were replaced every 1 or 2 months. The social and physical factors facilitating meth use in groups included social norms related to meth, having more free time, and the effects of meth. Most participants used meth to enhance friendly conversations.

There are always two, three people or more smoking ice together. Only meth addicts do it by themselves. People normally gather to talk. (Female, 42 years old, sex worker)

Typically, individuals did not just pay for their own consumption but took turns to purchase the drugs for each other. Some stated that they had never had to pay since they only did meth when their friends asked them to join.

The lockdown and social distancing did not seem to reduce meth use. Most participants reported little changes in their patterns of meth use. Having more free time during the lockdown meant more meth-smoking gatherings for some people.

[Before social distancing,] I was busy with my work. I didn’t think about [meth] or I wasn’t at home to join my friends. During the lockdown, we could go nowhere and had nothing to do. We called each other and went to a friend’s house. (Male, 27 years old, painter)

Outreach workers also observed increases in meth use among female sex workers:

In some places, before there was only one ice smoker, but now everyone smokes it. [...] People are more indebted to their patrons [because the patrons bought them drugs and food during the lockdown]. (FGD with peer outreach workers)

Five participants indicated the euphoric effects of meth and another reported meth-related hallucinations as physical risk factors that made them neglect the HCV, COVID-19 and other respiratory infection risks associated with these gatherings. When being asked how they perceived the risk related to sharing smoking pipes, some participants reported they did not even think about it:

My mind was on so many other things then. I was even hallucinating. (Male, 43 years old, unemployed)

Others said they were somewhat afraid of being infected with COVID-19 but they believed that they and their friends could not be infected:

Of course I’m afraid of COVID. But I trust them. (Female, 33 years old, sex workers)

On the other hand, the main protective factors against group meth use were COVID-related financial difficulties and their desire to hide their drug use from the family:

I used to do meth but now I don’t, because I’ve no money and if I do meth now, my parents will know and I don’t like it. I force myself to stay at home. (Female, 35 years old, unemployed)

### Sexual behaviors

Most male participants reported having lost interest in sex after years of drug use or as a side effect of methadone treatment. Compared to them, women were much more sexually active. Among the five women who reported selling sex occasionally or professionally, one had accepted unsafe sex in exchange for money during the lockdown. Risk factors were related to the COVID-19 epidemic.

Being afraid of COVID-19 infection during intimate transactions, many clients ceased visiting sex workers. The few clients who continued these visits paid sex workers less for the reason that clients earned less too. The curfew order requiring people to stay at home after 10 p.m. directly interfered with sex work since sexual transactions mostly occurred at nighttime. The resulting income loss made sex workers, especially those who were active heroin users, vulnerable, since they could no longer select their clients under the pressure of earning money for the next fix. One participant reported that she had to accept clients she deemed at-risk and had unsafe sex with them when they requested. The prospect of being in withdrawal was terrifying to her. She described her anxiety:

Only seeing them get into the room made me feel reassured. If they shook their head, I’d be nervous. Without a client, I’ll be dead. (Female, 38 years old, sex worker)

One protective factor was participants’ awareness of the HIV risk related to unsafe sex. In non-COVID times, most of these women insisted on consistent condom use with their clients. One woman accepted unprotected sex with her long-term elder clients but only after she had considered all potential risks. A participant said she almost hit a client when she found out he removed his condom during intercourse:

Who knows how many sex workers he’s gone with?! Around here I know three gals with AIDS. […] One or two hundred [thousand Dong] is not worth getting the disease. (Female, 33 years old, sex worker)

Thus, while participants were well aware of the HIV risk, financial challenges and the urge for heroin consumption might have forced them to resort to unsafe sex.

## Discussion

While PWID have been disproportionally affected by the COVID-19 epidemic, little is known about how their HIV/HCV risk behaviors change as a result of the epidemic, and the measures taken to contain it. This study is among the first studies exploring this topic in Vietnam. It highlights both risk and protective environmental factors influencing the risk behaviors of PWID in the unique context of Haiphong, a province with high harm reduction service coverage in the early phase of the COVID-19 epidemic in Vietnam. The findings herein are critical for harm reduction programs to support PWID during this global crisis.

The challenges that our participants experienced echo what has been found in other studies [1, 2]. Globally, PWID belong to the populations of low socioeconomic status who have been most disadvantaged during the COVID-19 crisis [1]. In Vietnam, as adult children, especially sons, cohabit with their parents to take care of them, most of our participants were living with their family during the lockdown. Thus, they could be more materially and emotionally supported than PWID in other parts of the world, who experienced homelessness and inadequate housing, making them vulnerable to COVID-19 infection and other life hazards [41, 42]. Nonetheless, Vietnamese PWID struggled financially to support themselves and their families. Since the social networks of PWID often consist of people of similarly low socioeconomic status [43], their usual support resources were also depleted under the impact of COVID-19. The epidemic made the experiences of social inequality more pronounced, as our participants observed that the middle class, with savings and the ability to work from home, are better protected from COVID-related financial crises than blue-collar workers. This resonates with Nanda’s writing about inequalities during the COVID-19 outbreak [1].

Unsafe injection did not seem to be an issue among PWID in Haiphong, thanks to the high awareness of participants regarding injection-related HIV/HCV risk. However, this might not be the case in provinces with lower harm reduction service coverage. Haiphong PWID did not seem to encounter challenges in accessing harm reduction services during the pandemic, unlike PWID in some European countries [3]. Pharmacies remained open during the lockdown, providing easy access to injection equipment. However, the punitive approach of the police in some areas might counteract the benefits of this, as it made PWID reluctant to get sterile syringes in public settings. This risk is real, given Vietnamese media having broadcast cases of drug users getting caught while they went out to buy drugs during the lockdown [44]. Moreover, as outreach workers indicated, it is important to consider when financial difficulties in buying heroin during the lockdown and social distancing period might facilitate group use of heroin, and with it, potential equipment sharing.

The common practice of group meth use and smoking equipment sharing is worrisome. This norm related to meth use has been reported in other studies among female sex workers, methadone patients, and people who use meth in general [22, 45, 46]. This practice carries with it a high HCV infection risk, as the HCV prevalence among PWID was nearly 90% [47], with low uptake of HCV care services in general [29]. Meth use could interfere with HIV treatment adherence and viral suppression, thereby increasing the likelihood of HIV transmission from people who use drugs to their sexual partners [48, 49]. Moreover, meth use facilitates tuberculosis transmission, a main cause of death among HIV-positive people who inject drugs [50]. Given that one reason for participants to use meth collectively was to cope with the boredom and stress related to the COVID-19 pandemic, it is likely that this practice will persist even after lockdown is lifted, since unemployment will likely continue to be an issue during the global recession [51].

The COVID-19 pandemic has created new challenges for sex workers in different parts of the world, especially those with untreated drug use disorders [9, 13, 52]. Our study shows that the financial pressure resulting from the containment measures could lead Vietnamese female sex workers to accept unsafe sex and risk their health. The link between drug use and acceptance of unsafe sex for more money has also been reported in a study among sex workers in Canada [53]. The livelihood of female sex workers during COVID-19 lockdowns is worrisome, given many had low education, no vocational training and no alternative jobs [54]. Moreover, compared to men, women who use drugs are more stigmatized and receive much less support from family and social services [55, 56]. The global crisis is exacerbating their vulnerabilities, and calls for action to be taken in order to minimize its devastating impact on this population.

## Recommendations

Our findings suggest harm reduction programs for PWID in provinces with high service coverage, like Haiphong, could consider focusing on meth use and on female sex workers during the COVID-19 epidemic. Regarding meth use, education and information about the risk related to group meth use and distribution of smoking tools would minimize HCV transmission risk. However, further efforts should be made to bring female sex workers into methadone treatment to help them deal with their financial stress. Harm reduction programs could be integrated into current social aid programs targeting populations of low socioeconomic status, in order to provide them with a comprehensive service package [57]. Outreach work should receive further investment, as outreach workers not only represent the bridge between people in need and treatment services, but also bring material support to those most in need [52]. Future studies should continue to document the living experiences of PWID and their risks as the epidemic and prolonged lockdown continue to affect most provinces in Vietnam.

## Limitations

The study would reflect more truthfully the experiences of participants if it had been conducted during the lockdown rather than after the lockdown had finished. The COVID-19 mitigation measures also made ethnographical work—a more appropriate method for the study question—unfeasible. While we intentionally over-recruited women, the number of female sex workers is small. Moreover, we were not able to recruit venue-based women who worked under the management of pimps. These participants might be living in different risk environments than our street-based participants. Finally, the COVID-19 situation in Vietnam has become much more severe than it was in 2020. PWID might currently experience greater challenges than our report shows.

## Conclusion

The COVID-19 pandemic disproportionally affected PWID and introduced new positive and negative influences on HIV/HCV risk behaviors. Most participants suffered from the financial impact of the epidemic mitigation measures. Unsafe injection might no longer be an issue among PWID in Haiphong, thanks to a combination of different factors including high awareness of injection-related HIV/HCV risk, and the availability of methadone treatment. However, group meth use and smoking equipment sharing as a common practice among PWID might elevate the risk for HCV, HIV and COVID-19 infection. Meth use as a means to cope with boredom and the stress related to COVID-19 might continue beyond the lockdown period. Female sex workers, especially those who were active heroin users, suffered most from COVID-related financial pressure and might resort to unsafe sex. These insights could orient harm reduction programs during this global crisis.

## Data Availability

The datasets generated during and/or analysed during the current study are available from the corresponding author on reasonable request.

## Abbreviations

DRIVE: DRug use and Infections in ViEtnam
FGD: Focus group discussion
PWID: People who inject drugs
